# High Intrinsic Expression of P-glycoprotein and Breast Cancer Resistance Protein in Canine Mammary Carcinomas Regardless of Immunophenotype and Outcome

**DOI:** 10.3390/ani11030658

**Published:** 2021-03-02

**Authors:** Michela Levi, Luisa Vera Muscatello, Barbara Brunetti, Cinzia Benazzi, Federico Parenti, Francesca Gobbo, Giancarlo Avallone, Barbara Bacci, Elisa Zambon, Paola Valenti, Giuseppe Sarli

**Affiliations:** 1 Department of Veterinary Medical Sciences, University of Bologna, Ozzano dell’Emilia, 40064 Bologna, Italy; michelalevi3@gmail.com (M.L.); luisaver.muscatello2@unibo.it (L.V.M.); b.brunetti@unibo.it (B.B.); cinzia.benazzi@unibo.it (C.B.); federico.parenti3@studio.unibo.it (F.P.); francesca.gobbo3@unibo.it (F.G.); giancarlo.avallone@unibo.it (G.A.); barbara.bacci@unibo.it (B.B.); 2 Ospedale Veterinario, I Portoni Rossi, Zola Predosa, 40069 Bologna, Italy; elisa.zambon@portonirossi.it; 3 Clinica Veterinaria Malpensa, Samarate, 21017 Varese, Italy; pvalenti.dvm@gmail.com

**Keywords:** P-glycoprotein, breast cancer resistance protein, multidrug resistance, canine mammary carcinoma, immunophenotypes

## Abstract

**Simple Summary:**

Multidrug resistance of neoplastic cells to chemotherapeutic drugs is a phenomenon mediated by several molecular mechanisms. Among these, P-glycoprotein (P-gp) and Breast Cancer Resistance Protein (BCRP) counteract the intracellular load of multiple drugs, preventing their efficacy. The basal (intrinsic) cellular expression can be further stimulated by drug exposure. P-gp and BCRP are a subject of intense investigation both in human and veterinary oncology since a better understanding of how their expression is distributed across different tumors allows planning alternative therapeutic strategies. In canine mammary carcinomas, a phenotypic classification similar to the one widely adopted for breast cancer is currently employed. For Basal- and Normal-like phenotypes, chemotherapy is still the main option. In this study, we observed that canine mammary carcinomas bear a high intrinsic expression of both P-gp and BCRP, regardless of their molecular phenotype, and their presence does not influence the outcome.

**Abstract:**

P-glycoprotein (P-gp) and breast cancer resistance protein (BCRP) are major actors in multidrug resistance (MDR) phenomenon in both human and canine mammary carcinomas (CMCs). The aim of this study was to investigate an association between the intrinsic expression of P-gp and BCRP compared to the immunophenotypes and outcome in CMCs. Fifty CMCs were evaluated at immunohistochemistry (IHC) for P-gp, BCRP, Estrogen receptor alpha (ER), Progesterone receptors (PR), Human Epidermal Growth Factor Receptor type 2 (HER2), basal cytokeratins 5/6 (CK5/6), Epidermal Growth Factor Receptor 1 (EGFR), and Ki67 proliferation index. P-gp and BCRP positive cases were, respectively, 52% and 74.5%, with a significantly higher expression of BCRP than P-gp. Five immunophenotypes were defined in 37 out of 50 CMCs: 9 (24.3%) Luminal A, 5 (13.5%) Luminal B, 9 (24.3%) HER2 overexpressing, 9 (24.3%) Triple-negative basal-like, and 5 (13.5%) Triple-negative non-basal-like. In all CMCs at least one marker was expressed. Follow-up data were available for 25 animals. The average cancer-specific survival was 739 ± 444 days. A number of CMCs bear a high expression of P-gp and BCRP but no significant association was found between their expression and the immunophenotypes, Ki67 index, the histological grade, and tumor-related death.

## 1. Introduction

Multidrug resistance (MDR) of neoplastic cells to multiple chemotherapeutic drug is a complex phenomenon which remains a major challenge in the treatment of cancer [1]. One of the most studied mechanisms that can lead to the development of chemoresistance is the overexpression of ATP-binding cassette (ABC) transporters that utilize ATP to efflux anti-cancer drugs across cellular membranes. ABC transporters comprise P-glycoprotein (P-gp/MDR1) encoded by the ABCB1 gene and breast cancer resistance protein (BCRP/mitoxantrone resistance protein) encoded by the ABCG2 gene which serve a variety of physiological functions including the protection of cells from potentially toxic xenobiotics. When P-gp and BCRP are overexpressed in neoplastic cells they pump chemotoxic compounds out of the intracellular compartment rendering cancer cells resistant to treatment [1]. Cancer resistance can be broadly classified into two categories, primary/intrinsic and secondary/acquired. Intrinsic drug resistance occurs prior to any given treatment and is due to the intrinsic expression and functionality of ABC transporters by neoplastic cells, which are therefore able to prevent the intracellular accumulation of drugs at induction chemotherapy. Acquired resistance develops after initial therapy when neoplastic cells build up the ability to express ABC-pumps after the first treatment [2,3,4]. In breast cancer research, intrinsic expression of P-gp and BCRP have been associated with poor prognosis, poor response to chemotherapy, amplification of the Human Epidermal Growth Factor Receptor type 2 (HER2) oncogene and hormonal negative status [5,6]. Nevertheless, the best responses to chemotherapy are seen in functionally negative tumors, i.e., in those tumors which do not present ABC-transporters capable of extruding compounds from the intracellular compartment, while the worst responses are more often registered in functionally P-gp/BCRP–positive tumors that bear ABC-transporters which pump anti-cancer drugs out of the cell preventing their action. P-gp and BCRP expression can be easily detected by immunologic techniques (i.e., IHC) and RNA hybridization methods (i.e., RT-PCR), but a main limitation of these methods is that the levels of expression may not correlate with the functional activity of the protein, which is best measured by flow cytometry assays or accumulation and efflux assays [7,8].

The expression of ABC-transporters P-gp and BCRP has been proven in both benign and malignant canine mammary tumors [9,10,11,12,13,14,15,16,17,18,19,20]. Overall, a higher intrinsic expression of P-gp and BCRP has been seen in aggressive histotypes and in carcinomas with higher histological stage and grade [9,14,15,18], but the role of these chemoresistance modulators in canine mammary carcinomas (CMCs) is still incipient.

The aim of this paper is to investigate the association between the immunohistochemical intrinsic expression of P-gp and BCRP with the molecular phenotypes of CMCs and tumor-related death.

## 2. Materials and Methods

### 2.1. Sample Selection, History and Histological Analysis

Samples were retrieved from the archive of the Department of Veterinary Medical Sciences, University of Bologna, of the Ospedale Veterinario “I Portoni Rossi” Bologna and of the Clinica Veterinaria Malpensa, Italy. Fifty formalin-fixed and paraffin-embedded tissue samples of mammary glands from 45 dogs were selected at the optic microscope based on the histopathological diagnoses of mammary carcinoma. The anamnestic and clinical data available from the archive, such as age, sex and neutering status, size of the tumor measured by the pathologist at the trimming of the surgical excised mass, presence of lymph node or systemic metastases and clinical stage (TNM system) were collected and the follow-up period was defined as 24 months between histologic diagnosis and last data collection. According to the reports, the dogs had not received chemotherapy at the time of biopsy/surgery. Cancer-specific survival (CSS) was defined as the period between surgery and tumor-related death, which was clinically outlined as spontaneous death or euthanasia due to tumor-related issues. Follow-up data were retrieved by phone calls to the referent veterinarian or to the owner of the dogs.

The original histologic diagnosis and histological grade was reviewed for each slide and updated, when necessary, according to the current histologic classification [21].

The study was conducted in accordance with guidelines and regulation and in compliance with the current national legal treatment of animal tissue samples.

### 2.2. Immunohistochemistry

A combination of 6 immunohistochemical markers: Estrogen Receptor alpha (ER), Progesterone Receptor (PR), HER2, Cytokeratins 5/6 (CK5/6), Epidermal Growth Factor Receptor 1 (EGFR), and Ki67, was used to define the immunophenotypes of CMCs as proposed for human breast cancer immunophenotypes [22]. P-gp and BCRP were assessed to establish the chemoresistance potential of CMCs. Formalin-fixed and paraffin wax-embedded tissues were sectioned (3 μm). The primary antibody types, dilutions, antigen retrieval methods, and tissues used as positive (internal and external) controls are reported in Table 1.

Prior to antigen retrieval, endogenous peroxidase was blocked by immersion in H_2_O_2_ 3% in methanol for 30′. Blocking of non-specific antigenic sites was achieved by incubating the slides in a solution of 10% goat serum and PBS for 30′ at room temperature. Slides were then rinsed with TRIS for reagent removal and were incubated overnight at 4 °C with the primary antibody diluted in a solution of 10% goat serum and PBS. The slides were rinsed in TRIS buffer and then incubated with secondary anti-mouse antibody (biotinylated goat anti-mouse immunoglobulins; Dako, Glostrup, Denmark) diluted 1 in 200 in 10% NGS in PBS. The reaction was revealed by a commercial streptavidin-biotin-peroxidase technique (ABC Kit Elite, Vector, Burlingame, CA, USA) and visualized with 3,3′-Diaminobenzidine in tablets (DAB chromogen/substrate kit; Diagnostic BioSystem, Pleasanton, CA, USA). Slides were counterstained with Harris’ hematoxylin and permanently mounted with DPX mountant.

External positive controls, and positive control internal to the examined tissue, when available, were examined. Corresponding negative control slides were processed in parallel by replacing the primary antibody with an isotype control non-reactive antibody, purchased at the same provider for each primary antibody.

All the markers except Ki67 were scored by semiquantitative evaluation of 10 representative high-power fields at the optical microscope.

P-gp and BCRP were considered positive when ≥20% and ≥10% of cells were labelled for P-gp and BCRP, respectively, as suggested by previous studies [14,18,23]. A further semiquantitative evaluation of the immunostaining was performed, and the positive carcinomas were assigned to 2 subgroups according to the percentage of positive cells: intermediate positivity (range of 20–50% of P-gp–positive cells; range of 10–50% of BCRP-positive cells) or high positivity (≥50% of P-gp–positive cells; ≥50% of BCRP-positive cells).

ER and PR immunolabelling were evaluated according to the consensus on stand-ard guidelines for hormone receptor assessment using immunohistochemistry [24,25] and the Allred score for the epithelial and myoepithelial component of each carcinoma was performed. According to the leading publication by Nguyen et al. (2018), a cut-off of ≥10% of positive nuclei in the epithelial component of carcinomas was adopted to assign hormones receptor positive status [26].

HER2 was scored based on the 2018 ASCO/CAP guidelines for the assessment of HER2 status in breast cancer. Carcinomas were considered HER2 positive only for a 3+ IHC score [26,27].

EGFR and CK5/6 were considered positive at a threshold ≥10% of positive cells as suggested by previous studies on canine mammary tumors [25,26].

A semiquantitative evaluation was performed for each marker, assessing the per-centage of positive cells at the optic microscope in 10 high power fields, representative of the histological subtype and grade of the carcinomas, as suggested by the literature [25,26].

Ki67 proliferation index was assessed by manual image analysis based on the number of positive nuclei among >500 neoplastic cells and expressed as a percentage (Image J software, National Institute of Health, Bethesda, MA, USA).

Cases showing no reactivity of the internal positive control, for the examined marker, presumably because of a deterioration of the antigen due to formalin fixation, were excluded.

Immunophenotypes were classified into five groups according to the scheme proposed for breast carcinomas by Nielsen et al. (2004) and translated to canine mammary carcinomas by Abadie et al. (2018) [22,28] and comprised:

- Luminal A: HER2 negative (HER2 0, 1+ or 2+); ER and/or PR positive; Ki67 < 33%.
- Luminal B: HER2 negative (HER2 0, 1+ or 2+); ER and/or PR positive; Ki67 ≥ 33%.
- HER2-overexpressing: HER2 positive (HER2 3+).
- Triple-negative basal-like: HER2 negative (HER2 0, 1+ or 2+); ER and PR negative; EGFR and/or CK5/6 positive.
- Triple-negative non-basal-like: HER2 negative (HER2 0, 1+ or 2+); ER and PR negative; EGFR and CK5/6 negative.

### 2.3. Statistical Analysis

Comparison between groups was analyzed by Chi square test with Yates correction. Correlations between categorical variables were analyzed using the Pearson χ^2^ test. Survival curves were computed using the Kaplan and Meier estimate and compared by log-rank test. For all statistical tests, a *p*-value < 0.05 was considered significant.

## 3. Results

All the data collected for this study are reported in Supplementary Materials Table S1.

### 3.1. Animal Data and Histopathological Characteristics of Tumors

The selected samples included 50 CMCs from 45 female dogs, 16 of which were spayed.

Age ranged from 4.7 to 13.6 years, with median age of 10 years and average value of 9.8 ± 2.6 years. Maximum tumor size was 50 mm diameter and minimum 5 mm, with an average value of 25.24 ± 12.24 mm.

Follow-up data at 24 months post-surgery were available for 25 animals, 13 of which were alive, and 15 were dead, 4 for causes unrelated to the mammary carcinoma. Minimum CSS was 59 days, corresponding to a 14-year-old spayed dog with inflammatory mammary carcinoma. Excluding dogs that died of causes other than mammary carcinoma, the average CSS was 739 ± 444 days.

Seven out of 14 carcinomas, for which information was available, presented lymph node metastasis at the time of diagnosis.

Four out of 16 carcinomas, for which information was available, presented systemic metastases (pulmonary and disseminated to multiple organs) at the time of diagnosis.

Fourteen carcinomas were classified according to their clinical stage (TNM system): 2 were stage I, 1 was stage II, 7 were stage IV and 4 were stage V.

Histologic subtypes, classified according to the most recent classification [21], were represented as follows: 15 complex carcinomas, 12 solid carcinomas (one of which with areas of adenosquamous differentiation), 6 tubulopapillary carcinomas, 6 mixed carcinomas, 3 comedocarcinomas, 2 tubular carcinomas, 2 invasive micropapillary carcinomas, 2 inflammatory carcinomas, 1 intraductal papillary carcinoma, and 1 lipid rich carcinoma.

There were 19 carcinomas that were histological grade I; 13 carcinomas were grade II; and 16 were grade III. Inflammatory carcinomas (2 in this caseload) were not graded by this system.

### 3.2. Immunohistochemistry

The results for each immunohistochemical marker are reported in Table 2.

**Figure 1:**
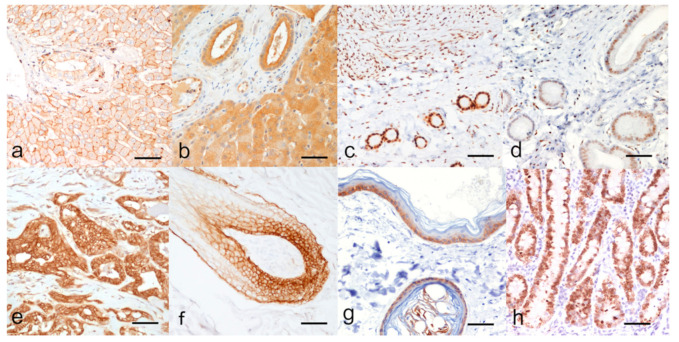
Immunohistochemical positive controls. (**a**) P-glycoprotein: strong immunolabeling of the plasma membrane and biliary canaliculi of hepatocytes and cholangiocytes in canine liver, (**b**) Breast Cancer resistance Protein: strong immunolabeling of the plasma membrane and cytoplasm of hepatocytes and cholangiocytes in canine liver, (**c**) Estrogen Receptor alpha: strong nuclear immunolabeling of endometrial glands, stromal cells and myometrium of canine uterus, (**d**) Progesterone Receptor: moderate to strong nuclear immunolabeling of endometrial glands and stromal cells of canine uterus, (**e**) Human Epidermal Growth Factor Receptor type 2: 3+ score, continuous, strong, membranous immunolabeling of luminal cells of canine mammary gland, (**f**) Epidermal Growth Factor Receptor type 1: strong membranous immunolabeling of epithelial cells of the follicular bulb of canine skin, (**g**) Basal cytokeratin 5 and 6: strong cytoplasmic immunolabeling of basal cells of the epidermis and follicular epithelium of canine skin, (**h**) Ki67 (MIB1 antibody): nuclear immunolabeling of cryptal enterocyte in canine small intestine. Indirect immunohistochemistry, magnification × 200, bar = 200 μm.

For each marker, the cases with inconsistent staining of the internal positive control were excluded from the study and comprised: 2 cases for P-gp, 3 cases for BCRP, 5 cases for ER, 14 cases for PR, 7 cases for EGFR, 4 cases for CK5/6, and 4 cases for Ki67. This allowed to summarize a molecular phenotype in only 37 out of the 50 CMCs available. Relevant pictures of the IHC external and/or internal positive CTR are reported in Figure 1.

**Figure 2:**
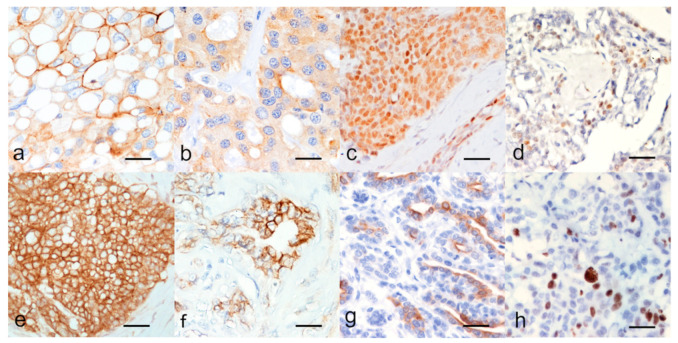
Immunohistochemical markers of CMCs. Positivity to (**a**) P-glycoprotein (P-gp strong membranous and weak/moderate multifocal cytoplasmic), (**b**) Breast Cancer Resistance Protein (BCRP membranous and multifocal cytoplasmic), (**c**) Estrogen Receptor alpha (ERα, nuclear), (**d**) Progesterone Receptor (PR, nuclear), (**e**) score 3 + for Human Epidermal Growth Factor Receptor type 2 (HER2, strong complete membranous labeling in all the neoplastic cells), (**f**) Epidermal Growth Factor Receptor type 1 (EGFR, membranous), (**g**) basal cytokeratin 5 and 6 (CK5/6, cytoplasmic), and (**h**) nuclear positivity to Ki67. Indirect immunohistochemistry, (**a**,**b**) magnification × 400, (**c**–**h**) magnification × 200.

P-gp immunolabelling was strong at the cellular membrane and cytoplasmic staining was found in a minority of positive neoplastic cells (Figure 2a). BCRP immunolabelling was membranous and cytoplasmic in most positive neoplastic cells (Figure 2b).

ER and PR immunostaining were nuclear in the luminal epithelial, and myoepithelial cells (Figure 2c,d, respectively) and were evaluated with the Allred score as reported in Supplementary Materials Table S2; the percentage of hormones receptor positive carcinomas are reported in Table 1. For both ER and PR, the most intense immunostaining was seen in the myometrium (positive external CTR, Figure 1c,d).

EGFR immunostaining was present multifocally to diffusely, with mild to intense positive reaction at the cell membrane mainly in neoplastic luminal epithelial cells and occasionally in myoepithelial cells (Figure 2f).

CK5/6 positivity was seen in myoepithelial and luminal epithelial cells with a cytoplasmic, discontinuous, multifocal, mild to intense staining (Figure 2g).

Ki67 immunostaining was observed at the nuclei (Figure 2h). Average Ki67 index was 25.69% ± 15.27; median 21.72%.

A total of 37 carcinomas out of 50 (74%) expressed at least one of the chemoresistance markers P-gp and/or BCRP. P-gp and BCRP were both expressed by the same carcinoma in 46% (23/50) of the tumors. BCRP alone was expressed in 12/50 carcinomas (24%), whereas P-gp alone was expressed only in 2/50 tumors (4%).

Considering the two markers investigated, BCRP positive cases were significantly higher than P-gp positive tumors (Chi square with Yates correction, *p* = 0.016).

We were able to classify 37 (74%) carcinomas by their immunophenotype. The remaining 13 (26%) carcinomas lacked consistent immunoreactivity for one of the markers required for the classification, possibly due to deterioration of the antigen caused by delayed or excessive long-lasting formalin fixation.

Carcinomas in our cohort were therefore subdivided as follows:

- Luminal A: 9 carcinomas (24.3%);
- Luminal B: 5 carcinomas (13.5%);
- HER2-overexpressing: 9 carcinomas (24.3%);
- Triple-negative basal-like: 9 carcinomas (24.3%);
- Triple-negative non-basal-like: 5 carcinomas (13.5%).

Luminal A, HER2-overexpressing and Triple-negative carcinomas were equally frequent in this caseload (9/37; 24%). An equal percentage of CMCs were of Luminal B and Triple-negative immunophenotypes (5/37, 13.5%). A significant association between immunophenotypes and Ki67 index (Spearman test, *p* < 0.05) was evident, with higher values in non-luminal non-HER2 overexpressing phenotypes (i.e., Triple-negative basal-like and non basal-like); while no correlation was revealed with grade (Spearman test, *p* > 0.05).

**Figure 3:**
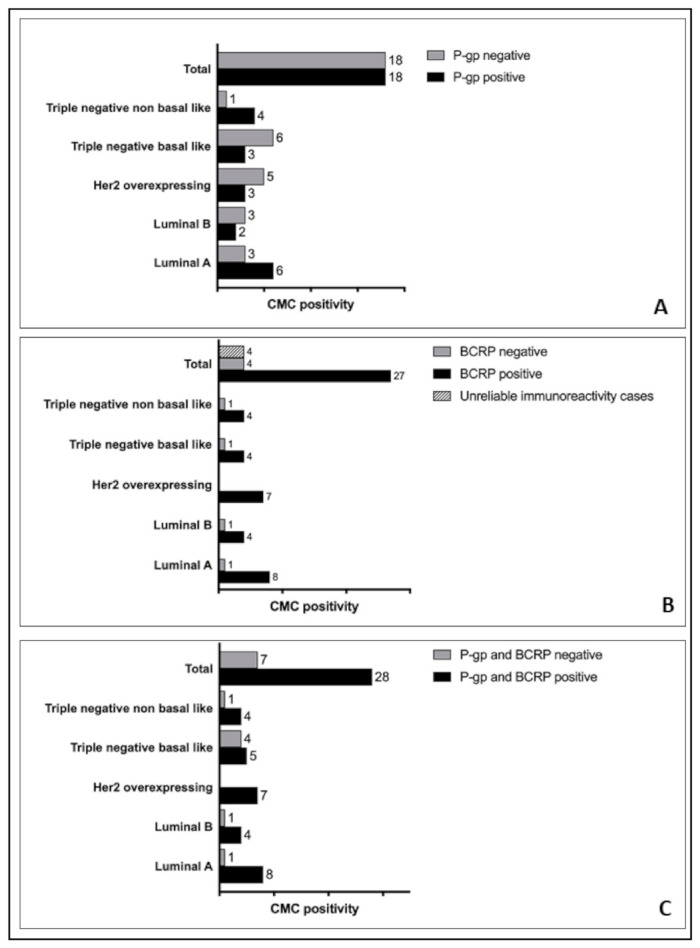
Graphic representation of the number of CMCs expressing P-gp (Panel (**A**)), BCRP (Panel (**B**)) or both markers (Panel (**C**)) in CMCs’ immunophenotypes.

P-gp, BCRP, and the coexpression of P-gp and BCRP in the different immunophenotypes is reported as graphs in Figure 3. The 2 HER2-overexpressing CMCs were not included in this group because of the lack of reliable BCRP expression in one CMC and of BCRP and P-gp expression in another CMC by IHC.

No significant association was found among the expression of P-gp or BCRP and the immunophenotypes (*p* > 0.05).

All HER2-overexpressing CMCs expressed at least one of the two chemoresistance markers (7/7), and no case was BCRP negative.

No statistical correlation was found among P-gp or BCRP expression or their co-expression and the Ki67 index (*p* > 0.05) or the histological grade (*p* > 0.05). The Ki67 index and the histological grade were significantly correlated (R = 0.67, *p* < 0.00001). The results of the correlation analyses are reported in Table 3.

**Figure 4:**
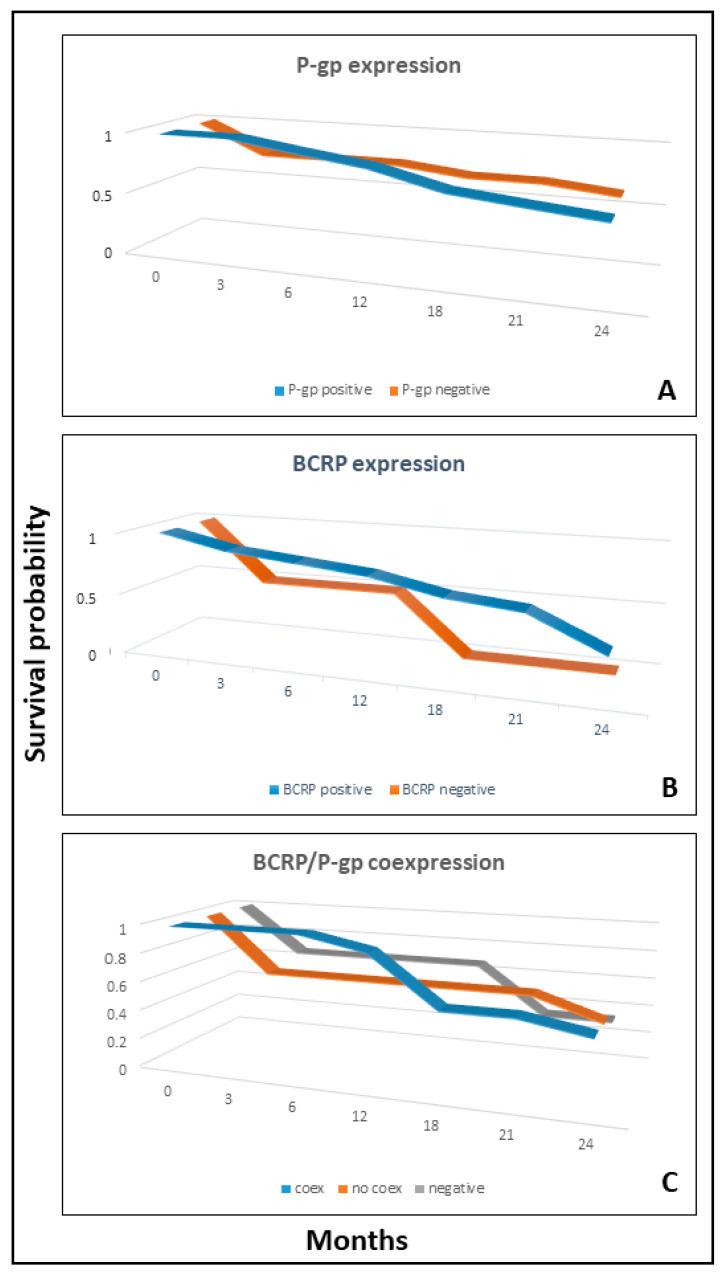
Survival curves of P-gp positive versus negative tumors (Panel (**A**)), the same comparison for BCRP (Panel (**B**)) and the comparison among groups expressing both markers (coex) vs. those expressing only one (no coex) or negative to both (Panel (**C**)). Survival analysis *p* > 0.05 in all comparisons.

Survival analysis did not reveal any differences in the outcome of female dogs bearing tumors positive to P-gp or BCRP singularly or combined with respect to female dogs bearing negative tumors (survival analysis *p* < 0.05, Figure 4).

## 4. Discussion

Breast cancer can be classified according to an immunohistochemical panel of molecular markers, which helps to predict the prognosis and guides the therapy in routine clinical practice [29,30]. Overall carcinomas are classified as Luminal if they express ER and/or PR while the amplification of the *HER2* gene drives an increased expression of HER2 that defines the HER2-overexpressing subtype [30]. Luminal and HER2 overexpressing carcinomas can benefit from specific therapies targeting their oncogenic pathways [31]. The risk of recurrence of Luminal carcinomas can be predicted more accurately with a further evaluation of the Ki67 labelling index and the expression of CK5/6 and EGFR [22,29]. Mammary carcinomas lacking the expression of ER, PR, and HER2 are known as Triple-negative breast cancers and are furtherly subclassified into basal-like carcinomas when expressing basal/myoepithelial markers: cytokeratins CK5/6, or CK14 and/or the EGFR [22,32]. Triple-negative carcinomas lacking the expression of basal markers are classified as Five-negative [29]. Currently no targeted therapy exists for Triple-negative and Five-negative subtypes, which bear the worst prognosis and require the administration of conventional chemotherapeutic protocols, leading to the emergence of chemoresistance in many patients [5,29,33]. Thus, there is a particular need to elucidate drug resistance mechanisms for this subtype, especially in triple negative breast cancer of which the core-basal subtype, which responds poorly to cytotoxic chemotherapy, has the worst prognosis [6].

Research on CMCs has successfully translated this human-based molecular classification to the bitch [25,28,34,35,36,37,38,39,40]. Even if major differences exist between human and canine mammary carcinomas with regard to the Luminal and HER2-overexpressing subtypes, the bitch has been confirmed as a useful spontaneous model for studying triple negative mammary carcinomas and the prognostic value of molecular subtyping has been demonstrated in both women and female dogs [28,41].

In this study, a high number of HER2-overexpressing carcinomas (24.3%) were detected. There is an ongoing longstanding controversy about the expression of HER2 in CMCs: some studies have reported significant levels of HER2 expression in CMCs [36,42,43], whereas others have questioned the feasibility of detecting HER2 overexpression by IHC [25,28,44].

Few studies have investigated a correlation between ABC-transporters overexpression in the subtypes of breast cancer. Increased expression of BCRP in invasive ductal carcinoma cells and its significant correlation with HER2 expression were found to be strongly correlated with tumor progression, invasion, and metastasis in two studies; no association with PR and ER status was found in one of these studies [45,46]. In vitro studies have shown a more frequent and intense BCRP expression in HER2-enriched mammary cancer cultured cells [47,48]. Other aggressive subtypes such as basal carcinomas had a high expression of BCRP/ABCG2 [6]. Interestingly, it has been suggested that BCRP/ABCG2 may affect the important role of cancer stem cells in drug resistance [49].

P-gp expression has been detected in a high percentage of breast cancers and was found to be increased after exposure to chemotherapeutic drugs (particularly those known to be P-gp substrates), and correlated with a worse response to treatment in both the adjuvant and neoadjuvant settings, but a direct role of P-gp as a cause of clinical drug resistance has not been adequately tested, even in breast cancer [7]. In one study, basal-like breast carcinomas were found to bear a higher expression of P-gp associated with the reduction or loss of estrogen receptor, progesterone receptor, and HER2 [50]. The expression of P-gp/MDR1 gene in cancer stem cells was found to be related with the molecular subtypes of breast cancer tissue: basal-like subtype and normal-like subtype had both significantly higher P-gp expression than both Luminal subtypes and HER2 overexpressing subtype, while HER2-overexpressing subtype has shown a significantly higher P-gp/MDR1 expression than Luminal subtypes [51].

At present there are no published studies investigating the expression of P-gp and BCRP in the immunophenotypes of CMCs. This led us to investigate the expression of the two most important ABC transporters that are associated with MDR, in the context of the different CMCs immunophenotypic subtypes. We hypothesized that this information could be relevant for therapeutic implications.

In the present study, a high percentage of CMCs was found to express at least one MDR marker, with half and 72% of carcinomas positive for P-gp and BCRP, respectively. The high intrinsic expression of chemoresistance markers P-gp and BCRP among malignant mammary carcinomas is a consistent finding in both human and canine mammary tumors, being reported in several studies [7,9,10,11,12,14,15,18]. Intrinsic expression of these membrane pumps in mammary glands, especially at the ductal epithelium, has been related to the physiological activity of this excretory organ and can be retained in neoplastic mammary cells [52,53].

Subgrouping mammary carcinomas into immunophenotypes was found not to be related to the expression of MDR markers in this study. This may be attributed to a loss of significance due to the small number of the caseload or to other factors. The most important can be that the retrospective collection of archived cases has various limits including, in this study, the unreliability of the IHC staining for some delicate antibodies (especially anti-PR) in formalin-fixed and paraffin-embedded tissue, an issue reported in other studies [54,55]. Despite this, an interesting finding in the present study was that all HER2-overexpressing CMCs expressed BCRP, which was consistent with studies regarding breast cancer; it could be related to unfavorable prognostic factors and suggests to administer the therapeutic protocol targeting HER2 avoiding conventional chemotherapeutic drugs that are BCRP substrates [47,48].

No significant association was found between P-gp, BCRP expression, and coexpression and any other variable investigated in this study, including Ki67 proliferation index, death due to the tumor, and histological grade. Histological grade, known to be associated with Ki67 [41], result confirmed also in this investigation, was previously found to be associated with P-gp expression in CMCs [14], even though opposite findings have been published [11]. This discrepancy could be related to the limited numerosity of the caseload in each mentioned study. However, it is likely that no correlation exists between P-gp and BCRP expression and immunophenotype of CMCs and no useful prognostic information can be extrapolated by a sole IHC analysis of their intrinsic expression. The biological significance of MDR associated pumps is better assessed with functionality assays [7]. In fact, *de novo* or intrinsic MDR occurs in a tumor when ABC transporters are expressed and functional in neoplastic cells before induction chemotherapy, whereas secondary or acquired expression of ABC transporters appear after the first chemotherapy treatment [4]. In this study we have been able to assess only the intrinsic (i.e., basal) expression of P-gp and BCRP in dogs which had not undergone chemotherapy at the time of surgical excision of the tumor. An explanation could be that ABC transporters other than P-gp and BCRP (i.e., MRP1/ABCC1, MRP3/ABCC3, MRP5/ABCC5, MRP6/ABCC6, MRP7/ABCC7 and/or ABCC11/MRP8) may have an important role in drug resistance in CMCs [6,10,19,20].

## 5. Conclusions

A relevant number of CMCs bear a high expression of P-gp and BCRP MDR markers, which could have therapeutic and prognostic implications. However, in the present study neither associations nor correlations were discovered between the intrinsic IHC expression of ABC transporters and immunophenotypes of CMCs or their relevance for survival. Nevertheless, BCRP is to a great extent expressed in CMCs and all HER2-overexpressing CMCs expressed at least one of the two chemoresistance markers.

## Figures and Tables

**Table 1:** Information about Immunohistochemistry (IHC) procedure.

Marker	Type, Clone	Supplier	Dilution Primary ab/Incubation	Ag Retrieval	Positive External CTR	Positive Internal CTR
P-gp	Mouse monoclonal anti–P-gp/CD243 (C494)	GeneTex International, Irvine, California	1:1500/ ON 4 °C	10′ Citrate pH6 MW:750 W	Canine liver	Lymphovascular endothelium
BCRP	Mouse monoclonal anti-BCRP (BXP-21)	Merck, Darmstadt, Germany	1:200/ ON 4 °C	10′ Citrate pH6 MW:750 W	Canine liver	Lymphovascular endothelium
ER alpha	Polyclonal anti-ER alpha	Thermo Fisher Scientific, Göteborg, Sweden	1:100/ ON 4 °C	10′ Citrate pH6 MW:750 W	Canine myometrium	Canine mammary gland
PR	Mouse monoclonal anti-PR (Ab-1)	Clabiochem/Merck KGaA, Darmstadt, Germany	1:50/ ON 4 °C	10′ Citrate pH6 MW:750 W	Canine myometrium	Canine mammary gland
HER2	Polyclonal anti-HER2 (A0485)	Dako, Glostrup, Denmark	1:200/ ON 4 °C	10′ Citrate pH6 MW:750 W	Canine mammary carcinoma HER2 score 3+	/
EGFR	Mouse monoclonal anti-EGFR Ab-10 (111.6)	NeoMarkers, Freemont, California	1:100/ ON 4 °C	15′ 37 °C Protease XIV 0.05% in PBS pH 7.5	Canine epidermis and hair follicles- basal layers	Canine epidermis and hair follicles- basal layers
CK5/6	Mouse monoclonal anti-CK5/6 (D5/16B4)	Zymed, South San Francisco, California	1:300/ ON 4 °C	15′ EDTA pH8 MW:750 W	Canine mammary gland- myoepithelium	Canine mammary gland- myoepithelium
Ki67	Mouse monoclonal anti-Ki67 (MIB-1)	Dako, Glostrup, Denmark	1:600/ ON 4 °C	20′ Citrate pH6 MW:750 W	Canine intestinal crypts	Hyperplastic canine mammary gland/hair follicles bulb

CTR, control; MW, microwave; ON, overnight.

**Table 2:** Immunohistochemical results: Percentage and number of samples scored as positive and negative for P-gp, BCRP, ER, PR, HER2, CK5/6, EGFR and Ki67 in the CMCs.

IHC Marker	Carcinomas *n*	%
P-gp total	48	
P-gp positive total (≥20% §)	25	52
P-gp (≥50% §)	10	20.8
P-gp (20–50% §)	15	31.2
P-gp negative (<10% §)	23	48
BCRP total	47	
BCRP positive total (≥10% §)	35	74.5
BCRP (≥50% §)	18	38.3
BCRP (10–50% §)	17	36.2
BCRP negative (<10% §)	12	25.5
ER total	45	
ER positive (≥10% §)	21	46.6
ER negative (<10% §)	24	53.4
PR total	36	
PR positive (≥10% §)	3	8.4
PR negative (<10% §)	33	91.6
HER2 total	50	
HER2 negative 0	6	12
HER2 negative 1+	22	44
HER2 negative 2+	13	26
HER2 positive 3+	9	18
EGFR total	43	
EGFR positive (≥10% §)	25	58
EGFR negative (<10% §)	18	42
CK5/6 total	46	
CK5/6 positive (≥10% §)	22	48
CK5/6 negative (<10% §)	24	52
Ki67 total	46	
Ki67 < 33% §	30	65
Ki67 ≥ 33% §	16	35

§ percentage of immunolabeled cells.

**Table 3:** Correlation of P-gp, BCRP expression or their coexpression with other variables of the study by Pearson test.

**Correlation Analysis**	**R**	***p* Value**
Immunophenotype		
P-gp positive CMCs	R = −0.0007	*p* = 1
BCRP positive CMCs	R = −0.296	*p* = 0.084
coexpression of P-gp and BCRP	R = −0.2998	*p* = 0.228
Ki67 > 33%		
P-gp positive CMCs	R = −0.1552	*p* = 0.315
BCRP positive CMCs	R = −0.0564	*p* = 0.7180
coexpression of P-gp and BCRP	R = −0.0345	*p* = 0.88
Histologic grade		
P-gp positive CMCs	R = −0.1753	*p* = 0.26
BCRP positive CMCs	R = 0.105	*p* = 0.502
coexpression of P-gp and BCRP	R = 0.0536	*p* = 0.814
Ki67 > 33%	R = 0.67	*p* < 0.00001

## Data Availability

All data generated or analyzed during this study are included in this published article and its supplementary information files. The raw datasets used and analyzed during the current study are available from the corresponding author on reasonable request.

## Supplementary Materials

The following are available online at https://www.mdpi.com/2076-2615/11/3/658/s1. Table S1: Excel file reporting all the data collected for this caseload study, Table S1: Excel file reporting the results of the Allred scoring system for Estrogen and Progesterone receptors, Table S2: Excel file reporting all the data collected for this caseload study.

## Abbreviations

ABC	Adenosine Triphosphate-binding cassette
CK 5/6	Basal cytokeratins 5/6
CMC	Canine mammary carcinoma
CTR	control
EGFR1	Epidermal Growth Factor Receptor type 1
ERα	Estrogen Receptor alpha
HER2	Human Epidermal Growth Factor Receptor type 2
IHC	immunohistochemistry
MDR	Multidrug resistance
PR	Progesterone Receptor
